# Electro-Acupuncture at Neiguan Pretreatment Alters Genome-Wide Gene Expressions and Protects Rat Myocardium against Ischemia-Reperfusion

**DOI:** 10.3390/molecules191016158

**Published:** 2014-10-09

**Authors:** Yan Huang, Sheng-Feng Lu, Chen-Jun Hu, Shu-Ping Fu, Wei-Xing Shen, Wan-Xin Liu, Qian Li, Ning Wang, Su-Yun He, Fan-Rong Liang, Bing-Mei Zhu

**Affiliations:** 1 Key Laboratory of Acupuncture and Medicine Research of Ministry of Education, Nanjing University of Chinese Medicine, No. 138, Xianlin Rd, Nanjing 210023, Jiangsu, China; E-Mails: hyan0915@sina.com (Y.H.); lushengfeng2013@gmail.com (S.-F.L.); shuping.fu@gmail.com (S.-P.F.); weixingshen@sina.cn (W.-X.S.); wliu@vet.upenn.edu (W.-X.L.); liqian20140603@163.com (Q.L.); hebei@263.net (N.W.); weixiaosoon@163.com (S.-Y.H.); 2 School of Information Technology, Nanjing University of Chinese Medicine, No. 138, Xianlin Rd, Nanjing 210023, Jiangsu, China; E-Mail: hucjhyl@126.com; 3 School of Acupuncture and Tuina, Chengdu University of Traditional Chinese Medicine, No.1166, Liutai Avenue, Wenjiang District, Chengdu 611137, Sichuan, China; E-Mail: acu973@126.com

**Keywords:** electro-acupuncture pretreatment, myocardial ischemia-reperfusion, RNA-seq, gene expression profiling, pathway

## Abstract

This study investigated genome-wide gene expressions and the cardioprotective effects of electro-acupuncture pretreatment at the PC6 Neiguan acupoint on myocardial ischemia reperfusion (I/R) injury. Male SD rats were randomly divided into four groups: sham operation (SO), I/R, electro-acupuncture at the PC6 Neiguan acupoint pretreatment (EA) and electro-acupuncture at non-acupoint pretreatment (NA). Compared with the I/R group, the survival rate of the EA group was significantly increased, the arrhythmia score, infarction area, serum concentrations of CK, LDH and CK-Mb and plasma level of cTnT were significantly decreased. RNA-seq results showed that 725 genes were up-regulated and 861 genes were down-regulated under I/R conditions compared to the SO group; both EA and NA reversed some of these gene expression levels (592 in EA and 238 in NA group). KEGG pathway analysis indicated that these genes were involved in multiple pathways, including ECM, MAPK signaling, apoptosis, cytokine and leukocyte pathways. In addition, some pathways were uniquely regulated by EA, but not NA pretreatment, such as oxidative stress, cardiac muscle contraction, gap junction, vascular smooth muscle contraction, hypertrophic, NOD-like receptor, and P53 and B-cell receptor pathways. This study was first to reveal the gene expression signatures of acute myocardial I/R injury and electro-acupuncture pretreatment in rats.

## 1. Introduction

Acute myocardial infarction (AMI) is a major cause of morbidity. Primary percutaneous coronary intervention (PPCI), as the most effective therapy for reducing acute myocardial injury, can preserve cardiac function and improve clinical outcomes due to its timely and effective reperfusion. However, the process of reperfusion, so-called myocardial ischemia reperfusion, could possibly result in lethal arrhythmias and cardiomyocyte death [1]. Therefore, new modalities for preventing myocardium against I/R injury are required for the optimal treatment of AMI.

Acupuncture has been practiced in China for over two thousand years, and is widely accepted as a clinical treatment for several diseases across the world. It has been documented that acupuncture at the PC6 Neiguan acupoint can improve symptoms of angina and palpitation, and can enhance left cardiac function in coronary heart disease [2,3]. Electro-acupuncture pretreatment can alleviate cardiac I/R injury in adult patients undergoing heart valve replacement surgery, by reducing the level of serum cardiac troponin I and the inotrope score, and shortening intensive care unit stay time [4]. However, the underlying molecular mechanisms by which Neiguan could be protective to myocardial I/R injury remain to be explored.

Although many studies have looked at the systemic effects of acupuncture on myocardial I/R injury, no research has been conducted on the response to electro-acupuncture pretreatment at the transcriptomic level of functional genes [5]. In this study, the potential protective effects of electro‑acupuncture at the PC6 Neiguan acupoint were investigated on rat myocardial I/R injury. Furthermore, we employed RNA sequencing (RNA-seq) to generate gene expression profiles for the rat hearts subjected to myocardial I/R injury with or without electro-acupuncture treatment at the PC6 Neiguan acupoint or non-acupoint. Our data uncovered distinct gene expression signatures among the I/R, EA and NA groups. Our study provided useful insights for understanding the molecular mechanisms underlying I/R, and the protective effects of pretreatment with electro-acupuncture at PC6 or non-acupoint on myocardial I/R injury by dramatically altering gene expression.

## 2. Results and Discussion

### 2.1. Electro-Acupuncture at the PC6 Neiguan Acupoint Pretreatment Effectively Protected Myocardium from I/R Injury

**Figure 1:**
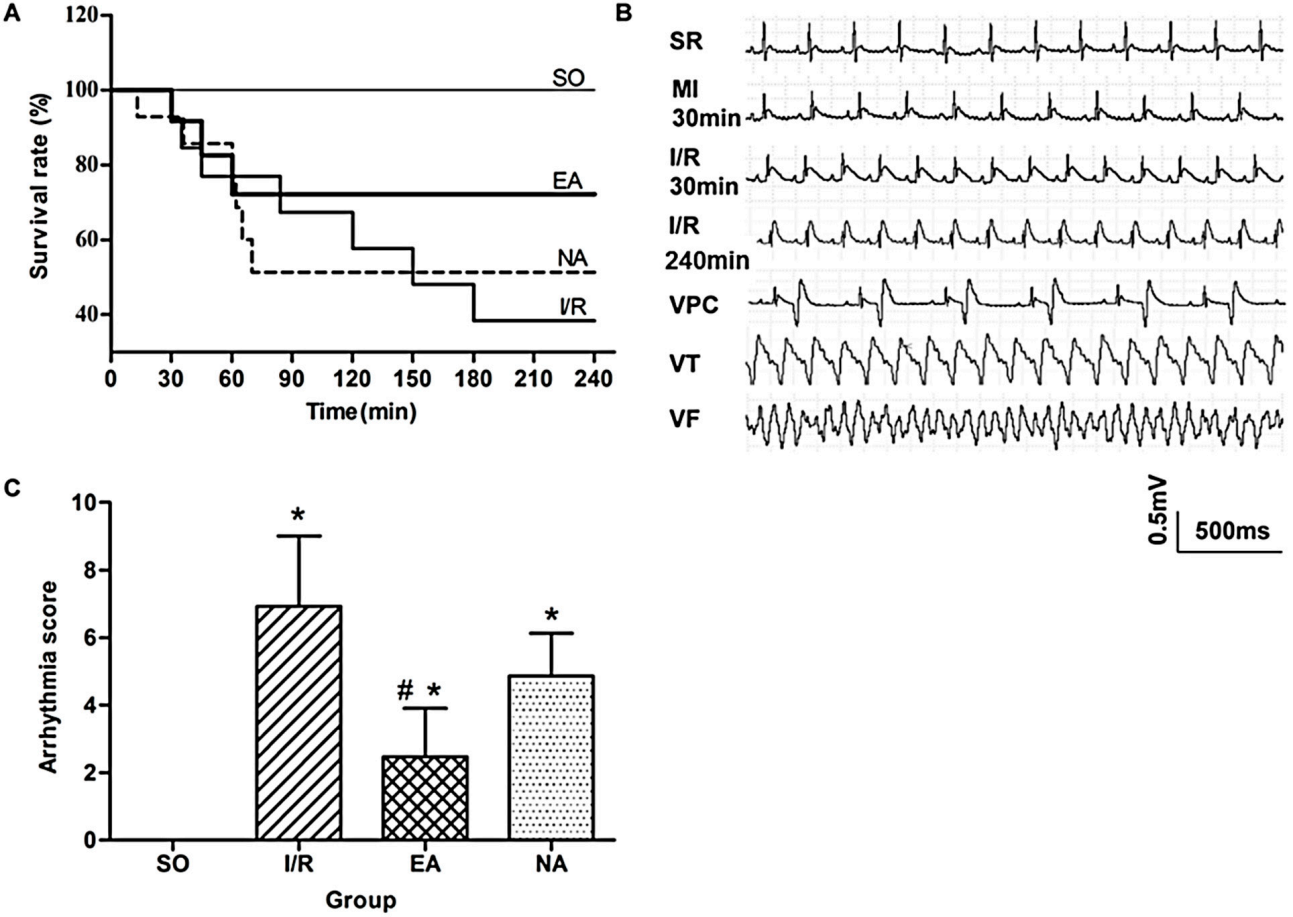
Anti-arrhythmic effect of EA. (**A**) Kaplan-Meier survival plots in 4 groups: SO (*n* = 15), I/R (*n* = 15), EA (*n* = 15), and NA (*n* = 15); (**B**) Representative ECG records measured from SO, I/R, EA, and NA rats. MI, myocardial ischemia; SR, sinus rhythm; VPC, ventricular premature contraction; VT, ventricular tachycardia; VF, ventricular fibrillation; (**C**) Quantitative analysis for arrhythmia scores. * *p* < 0.05, compared with SO group, #, *p* < 0.05, in comparison with I/R group (*n* = 15 for each group).

To investigate the effects of electro-acupuncture at the PC6 Neiguan acupoint pretreatment on myocardial I/R injury, we first observed changes in rat survival rates (Figure 1A). Mortalities were reduced from 9/15 in the I/R group to 7/15 in the NA group, compared with 0/15 in the SO group. Unsurprisingly, EA pretreatment much more drastically reduced the death of rats to 4/15 (Figure 1A). The ECG records (Figure 1B) show that the ST segments were visibly elevated after 30 min of ischemia, and first 30 min and 240 min after reperfusion. The occurrence of lethal arrhythmias, including VPC, VT and VF, was evaluated during 30 min of the ischemia and the first 30 min of reperfusion. Arrhythmia scores were assessed based on the system created by Curtis and Walker [6]. Compared with the SO rats, arrhythmia scores increased significantly in the I/R rats, but decreased in the EA rats (6.93 ± 2.08 in I/R group *vs*. 2.47 ± 1.44 in I/R group, *p* < 0.05) (Figure 1C).

**Figure 2:**
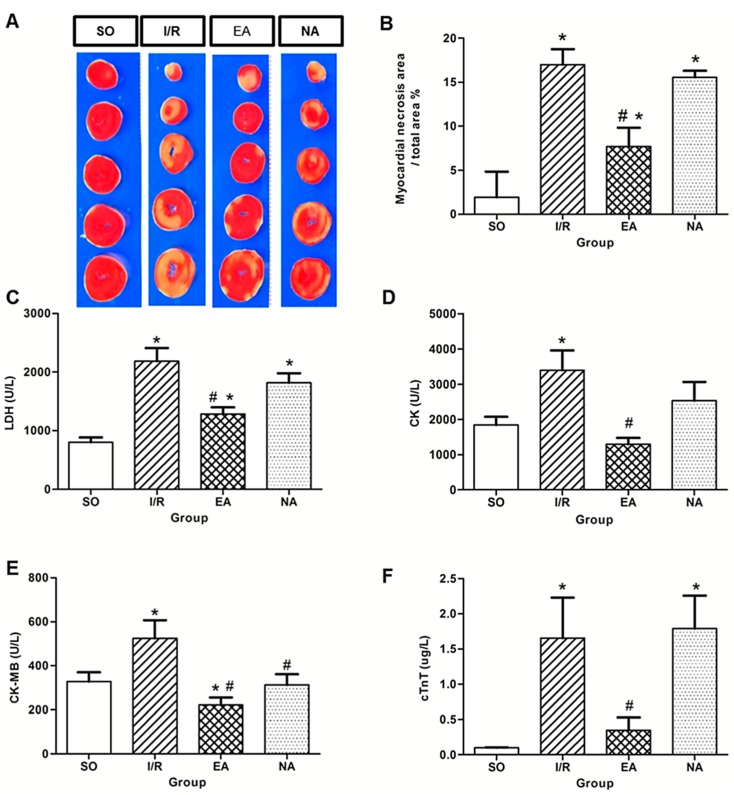
Pretreatment with EA reduced infarct size and myocardial enzymes. (**A**) Representative TTC staining of the hearts harvested from SO, I/R, EA, and NA groups; (**B**) Infarct size percentage measured by Image J. * *p* < 0.05, compared with SO group, # *p* < 0.05, in comparison with I/R group. *n* = 5 in SO, *n* = 4 in I/R, *n* = 5 in EA, *n* = 5 in NA; (**C**–**F**) Effect of EA pretreatment on myocardial enzyme levels, including LDH, CK, CK-Mb, and cTnT released into serum and plasma after myocardial ischemia reperfusion in rats. * *p* < 0.05, compared with SO group, # *p* < 0.05, in comparison with I/R group. U/L, Units/Litre. *n* = 15 in SO, *n* = 6 in I/R, *n* = 11 in EA, *n* = 8 in NA.

Following 4 h of reperfusion, the infarct size was determined by TTC staining. There was a significant difference in infarct size between the SO and the I/R rats (Figure 2A,B). EA markedly reduced myocardial injury size (7.69% in EA group *vs*. 17% in I/R group), whereas NA provided no effective protection. The measurement of the serum levels of myocardial specific enzymes after 240 min reperfusion, including lactate dehydrogenase (LDH), creatine kinase (CK), creatine kinase Mb (CK‑Mb), and cardiac troponin T (cTnT) confirmed cardioprotective effect of EA. The presence of these proteins in plasma serves as markers of myocardial and muscular damage. The levels of LDH, CK, CK-Mb, cTnT were significantly increased after I/R, EA significantly decreased the enzyme concentrations of CK (68.27%), CK-Mb (58.55%), LDH (58.35%), and cTnT (78.84%) compared to the I/R group (*p* < 0.05) (Figure 2C–F).

Acupuncture is increasingly being accepted as an alternative medical therapy worldwide due to its frank effectiveness. Our previous study has shown that acupuncture at PC6 protects ischemic injury in rats by improving angiogenesis [7]. In the present study, we investigated a novel cardioprotective role of acupuncture at the PC6 Neiguan acupoint when it was applied prior to injury, referred to as electro-acupuncture pretreatment. Our results were consistent with other study [8]. Electro-acupuncture pretreatment at the PC6 acupoint did protect against myocardial I/R injury by optimally reducing infarct size (Figure 2A,B) and arrhythmia score (Figure 1C), and increasing survival rate (Figure 1A). Meanwhile, the protective effect of electro-acupuncture pretreatment against reperfusion injury was confirmed by decreasing levels of LDH (Figure 2C), CK (Figure 2D), CK-MB (Figure 2E), and cTnT (Figure 2F).

### 2.2. Electro-Acupuncture at the PC6 Neiguan Acupoint Pretreatment Altered Gene Expressions Genome-Widely

Our study confirmed the cardioprotective effects of electro-acupuncture on myocardial I/R injury. We then investigated its possible molecular mechanisms by extracting RNAs from heart tissues and completing gene expression profiling for the different groups by using RNA-seq analysis. Sample quality for RNA libraries were confirmed by a multidimensional scaling (MDS) analysis (Supplementary Figure S1). Our results showed that, compared to the SO group, 1586 genes were differentially expressed in the I/R group; out of these 1586 genes, 861 genes were down-regulated, and 725 genes were up‑regulated. Electro-acupuncutre pretreatment down-regulated 649 genes and up‑regulated 385 genes compared with the I/R group, whereas, NA pretreatment down-regulated 355 genes and up-regulated 364 genes compared with the I/R group (Table 1). 

**Table 1:** DEGs across four groups.

DEGs	I/R:SO	EA:I/R	NA:I/R
Up regulated	725	385	364
Down regulated	861	649	355
Total	1586	1034	719

**Figure 3:**
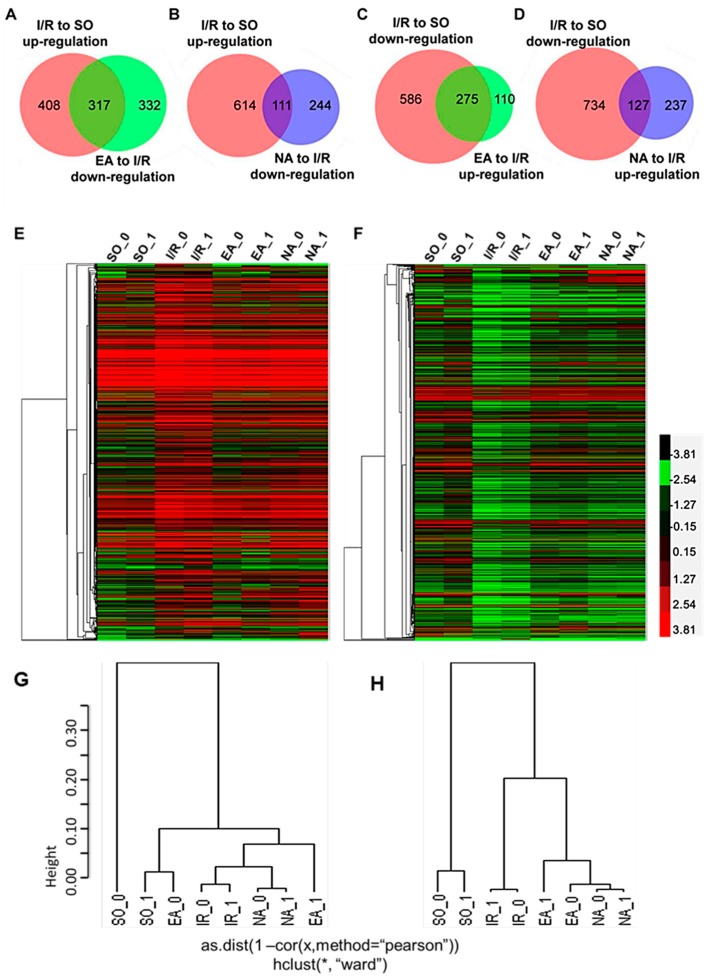
Venn diagrams and clustering analysis of RNA-seq results. (**A**–**D**) Venn diagrams were drawn based on our RNA-seq data sets. Red circles indicate the numbers of genes up- or down-regulated in I/R group (*vs*. SO group); green circles in (A,C) represent the numbers of down- or up-regulated genes in the EA group (*vs*. I/R group). Blue circles in (B,D) represent the numbers of down- or up-regulated genes in the NA group (*vs*. I/R group). The heatmaps were created with up-regulated (**E**) and down‑regulated (**F**) DEGs in myocardial I/R injury. Expression levels of up-regulated and down-regulated genes are represented in red or green colors, respectively. Two dendrograms of hierarchical clustering of 8 samples using the 725 up-regulated genes in I/R group (**G**) and the 861 down-regulated genes in the I/R group (**H**).

To further look into the differences in gene expression in these groups, we analyzed overlaps of the genes with Venn Diagrams (Figure 3A–D). We found that in the 725 up-regulated genes from the I/R group, 44% (317) of the genes were down-regulated by EA pretreatment, and in the 861 down-regulated genes from the I/R group, 32% (275) of the genes were up-regulated by EA pretreatment (Figure 3A,B). We noted that NA pretreatment reversed I/R-induced changes of gene expression as well, but the numbers of differentially expressed genes (DEGs) were far less than those in the EA group; only 15% (111) genes of the 725 up-regulated genes in I/R group were down-regulated, and 15% (127) genes of the 861 down-regulated genes in I/R group were up-regulated by NA pretreatment (Figure 3C,D).

We further analyzed the expression patterns by clustering of RNA-seq data. Cluster 3.0 program and the free software Treeview) [9] were used to display the genome-wide expression patterns with the Log10 ratio of four groups [10]. The heatmaps were created with the pathologically up-regulated genes (Figure 3E) and down-regulated genes (Figure 3F) in the I/R group. They also illustrate the alteration patterns of these genes by EA and NA pretreatments. The red color depicts higher expression, and the green color refers to lower expression of genes. Ward linkage method was used for the hierarchical clustering. 

The resulting dendrograms are shown in Figure 3G,H. From the MDS (Supplementary Figure S1), we could find that EA and SO were located in the upper right quadrant. Together with the data shown in Figure 3E,F, it suggests that the EA and SO groups were more similar to each other, but not to the I/R and NA group. This analysis is a visual confirmation that electro-acupuncture pretreatment regulates more genes that were pathologically changed in the I/R injury hearts compared with NA pretreatment.

#### 2.2.1. The Most Differentially Expressed Genes

To provide more detailed information, the top 30 differentially expressed genes (DGEs) with the highest fold changes in IR group are listed in Table 2A,B. The changes resulted from EA and NA pretreatment are included in these tables as well. Compared with the normal rats (SO), some genes were detected with highly up-regulated expression in the condition of I/R (Table 2A). Obviously, 23 genes out of the top 30 genes were reversed by EA, while there were only 12 genes down-regulated by NA. Five genes (Camp, Vdac3, Hmox1, Cyr61 and Mt2A) were not regulated by either EA or NA. Hspa1b, encodes stress-inducible 70-kDa heat shock proteins (HSP70) that protect cells from insults such as ischemia and reperfusion-induced damages in the heart and kidney [11,12]. EA, but not NA pretreatment, down-regulated its expression to normal levels with a FC (Log2) of −1.12. Tnfrsf12a, a member of TNF family was up-regulated considerably with 2.67 of FC (Log2) by I/R in our study and largely reversed by EA but not NA (Table 2A). This gene encodes a 14-kDa protein (Fn14, TNFrsf12a or Tweakr), which had been shown to play a critical role in cardiac remodeling. A study by Chorianopoulos and colleagues has identified the expression and regulation of Fn14 in cardiomyocytes and in experimental myocardial infarction [13]. Another study also provided evidence, by using an ischemia-reperfusion injury model through hemorrhage and a supraceliac cross-clamp followed by 6h resuscitation, that Tnfrsf12a was involved in apoptosis/cell death and angiogenesis/vascular development [14]. Our results showed a marked down-regulation of Tnfrsf12a gene with a FC (Log2) of −2.39 in the EA group (Table 2A) but no change in the NA group. The regulation by EA for the above genes possibly indicates reduced I/R injury when electro-acupuncture is applied to the PC6 Neiguan acupoint in the rats as a pretreatment approach. In contrast, the expression levels of Lcn2 (lipocalin-2) and Nr4a1 were not regulated significantly by EA, but down-regulated by NA in the present study (Table 2A). Lcn2 improved the functional recovery of isolated mice hearts subjected to I/R, which is associated with restoration of mitochondrial function and phospholipids remodeling [15]. Nr4a1, is reported to be an immediate-early response gene during IR injury and mediates cardiomyocyte apoptosis [16]. The down‑regulation of Lcn2 and Nr4a1 by NA, but not EA, might suggest that those genes were not functionally related to I/R injury in this particular model. Aside from the above genes that were differently modulated by EA and NA, some other genes, whose protective function against I/R injury had also been well documented previously, were highly expressed in the I/R hearts in this study and were regulated similarly by both EA and NA pretreatments. These genes include, Fos, Vdac3, and Trh (Table 2A). Co-induction of C-fos and HSP70 has been observed in the myocardium under hemodynamic stress [17]. As immediate-early genes, they have been investigated by numerous studies and showed to be protective to ischemia-reperfusion injury in the heart, brain, kidney, and liver [18,19,20,21]. Mitochondrial voltage-dependent anion channel (VDAC) is a protein at the crossroads of metabolic and survival pathways. The role of VDAC1 and of the other isoforms VDAC2 and VDAC3 in cell death is multi-faceted and complex [22,23,24]. Binding of anti-apoptotic Bcl-2 and BclxL to VDAC1 (with resulting inhibition of porin) has an anti-apoptotic action [25]. In contrast, Bax‑induced cytochrome c release was observed in mitochondria isolated from all WT, VDAC1-, VDAC3-, and VDAC1/VDAC3-null cells [26], suggesting a controversial consequence for Vdac3s function on apoptosis. Trh gene encodes a member of the thyrotropin-releasing hormone family. The expression level of Trh is very low in normal heart tissues but increases significantly under I/R injury in our study, and both EA and NA pretreatment were able to down-regulate its expression to the normal level (Table 2A). Thyroid hormones (THs) are reported to be critical for fetal cardiomyocyte maturation [27]. A previous study has shown that thyrotropin-releasing hormone receptor (TRHR) gene participates in the etiopathogenesis of essential hypertension [28], but the direct function of Trh on I/R injury is still unknown. The similarity of regulation patterns of EA and NA on the above genes might be due to the non-specificity of acupoint and non-acupoint on the genes whose functions were not specifically related to the protection of acupuncture to this particular I/R model.

**Table 2:** The top 30 differentially expressed genes with a log2 (FC) > |±1|.

A: The Top 30 Up-Regulated Genes in I/R				
Gene Name	Description	I/R *vs*. SO	EA *vs*. I/R	NA *vs*. I/R
Hspa1b	heat shock 70 kD protein 1B	3.58	−1.12	0.97
Mir3074	microRNA 3074	3.1	−3.13	0.6
Kbtbd5	kelch repeat and BTB (POZ) domain containing 5	3.04	−2.87	−0.39
Fam46b	family with sequence similarity 46, member B	2.7	−1.44	−1.15
Hist1h1d	histone cluster 1, H1d	2.7	-	−1.85
Tnfrsf12a	tumor necrosis factor receptor superfamily, member 12A	2.67	−2.39	−0.43
Fos	FBJ osteosarcoma oncogene	2.57	−1.18	−1.15
Atf3	activating transcription factor 3	2.5	−1.31	−0.72
Has1	hyaluronan synthase 1	2.47	−1.83	−0.31
Camp	cathelicidin antimicrobial peptide	2.46	−0.06	1.3
Mir3556b	microRNA 3556b	2.37	-	-
Vdac3	voltage-dependent anion channel 3	2.28	0.3	−0.14
Trh	thyrotropin releasing hormone	2.24	−2.14	−2.75
Hmox1	hemeoxygenase (decycling) 1	2.23	−0.78	0.7
Cyr61	cysteine-rich, angiogenic inducer, 61	2.22	−0.98	−0.09
Pgf	placental growth factor	2.21	−1.81	0.32
Slc7a5	solute carrier family 7, member 5	2.21	−1.33	−0.08
Klk12	kallikrein related-peptidase 12	2.09	−1.5	−1.81
Sphk1	sphingosine kinase 1	2.09	−1.74	−0.72
Cblc	Cbl proto-oncogene C, E3 ubiquitin protein ligase	2.03	−1.84	−2.24
Nr4a1	nuclear receptor subfamily 4, group A, member 1	2	−0.72	−2.35
Numbl	numb homolog (Drosophila)-like	2	−1.58	−1.14
Mt1a	metallothionein 1A	1.97	−1.77	−0.44
Flnc	filamin C, gamma	1.95	−1.69	0.14
Lcn2	lipocalin 2	1.95	−0.84	−3.48
Gal	Galanin	1.94	−1.32	−1.67
Alox15	arachidonate 15-lipoxygenase	1.94	−1.74	−0.66
Akr1b8	aldo-keto reductase family 1, member B8	1.94	−1.19	−0.41
Mt2A	metallothionein 2A	1.9	−0.8	0.62
Cnot3	CCR4-NOT transcription complex, subunit 3	1.89	−1.47	−1.13
LOC367975	unknown	−5.86	2.19	2.72
Mir145	microRNA 145	−5.32	-	-
Klre1	killer cell lectin-like receptor family E member 1	−4.58	4.27	4.8
Ky	kyphoscoliosis peptidase	−3.62	0.78	0.42
Clecsf6	C-type lectin domain family 4, member A	−3.45	2.68	1.56
RGD1306750	unknown	−3.21	2.09	1.78
Cybb	cytochrome b-245, beta polypeptide	−2.83	2.38	2.45
Ptplad2	protein tyrosine phosphatase-like A domain containing 2	−2.78	1.91	0.86
Cyp2e1	cytochrome P450, family 2, subfamily E, polypeptide 1	−2.74	2.82	−0.38
Sh2d1a	SH2 domain containing 1A	−2.71	2.4	2.73
Tas2r120	taste receptor, type 2, member 120	−2.6	1.95	0.92
Epsti1	epithelial stromal interaction 1	−2.57	1.84	1.74
C6	complement component 6	−2.56	1.35	1.54
F13a1	coagulation factor XIII, A1 polypeptide	−2.54	1.9	1.17
Bex4	brain expressed, X-linked 4	−2.5	1.31	1.05
Tfec	transcription factor EC	−2.5	2.58	2.16
Msr1	macrophage scavenger receptor 1	−2.48	2.15	3.17
Ptprc	protein tyrosine phosphatase, receptor type, C	−2.47	0.3	1.93
Clec4a2	C-type lectin domain family 4, member A2	−2.45	2.31	1.75
Cd69	CD69molecule	−2.42	1.89	1.04
Agr2	anterior gradient 2	−2.41	0.25	1.26
Igsf6	immunoglobulin superfamily, member 6	−2.41	1.73	2.51
Lilra5	leukocyte immunoglobulin-like receptor, subfamily A, member 5	−2.38	2.06	−0.23
Klri1	killer cell lectin-like receptor family I member 1	−2.34	2.18	1.73
Ubd	ubiquitin D	−2.32	0.46	0.98
Ahr	aryl hydrocarbon receptor	−2.32	1.78	1.33
Ccr2	chemokine (C-C motif) receptor 2	−2.31	2.52	2.7
Pkhd1l1	polycystic kidney and hepatic disease 1-like 1	−2.31	2.03	1.45
Mpeg1	macrophage expressed 1	−2.29	2.07	2.25
Dock11	dedicator of cytokinesis 11	−2.28	1.62	1.19

Specified values of log2 (log2 FC|±1) refer to SO in I/R, and to I/R in EA and NA. Minus refers to down-regulation; “-” represents data not available.

**Figure 4:**
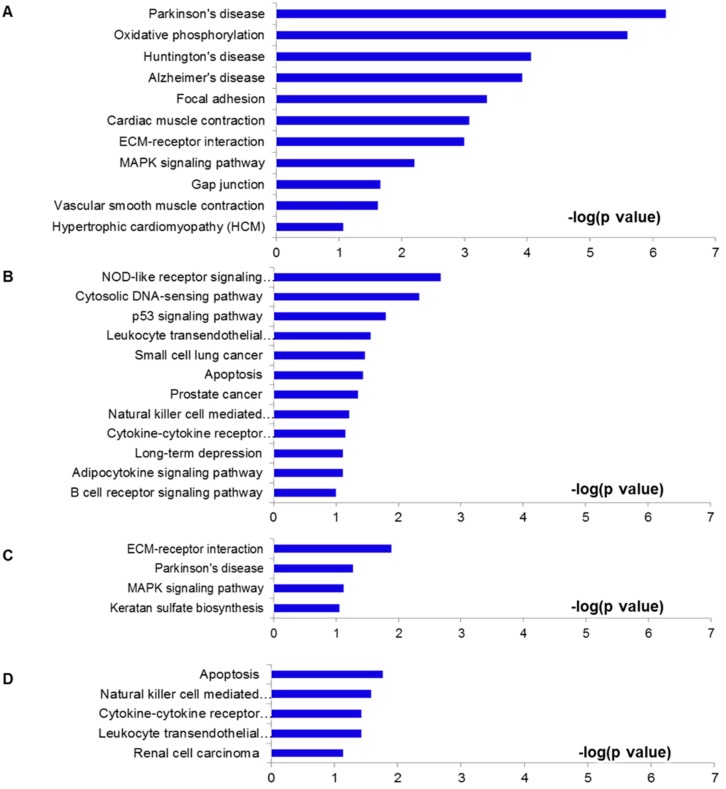
Pathways analysis. (**A**–**D**) Pathways of these overlap genes were analyzed using KEGG pathway program, based on individual over lapped gene numbers shown in Figure 3. (A) was drawn from the 317 genes shown in Figure 3A; (B) from the 275 genes in Figure 3C; (C) from the 111 genes in Figure 3B; (D) from the 127 genes in Figure 3D.

Table 2B shows the top 30 down-regulated genes in I/R hearts and the changes resulted from EA and NA pretreatment. Obviously, 25 genes out of the top 30 genes were reversed by EA, and 23 genes by NA. Ky and Ubd were not reversed by either EA or NA. Among these top genes, we noted that some of them (LOC367975 and RGD1306750) have unknown functions, and many of them are related to the immune system. For example, a killer cell lectin-like receptor family E member 1 (Klre1) is mapped to the NK gene complex and inhibits natural killer cell cytotoxicity and was reported to be up‑regulated in acute ischemic brain [29]. Msr1, namely SR-A, is critical for normal healing after MI, and Msr1 appeared to be strong candidate genes for Civq4, which modulates infarct volume after ischemic stroke [30]. Recent study showed that TNF-α secretion was enhanced in SR-A^−/−^mice, and could be responsible for the increased MMP activity and augmented risk of MI [31]; SR-A has a role in the induction of innate immunity and plays a central role in cerebral ischemia/reperfusion injury [32]. The present study is not sufficient to explain why those genes whose expressions were down-regulated in I/R, but were up‑regulated by EA and NA treatments closely at the same extent. Combined with the further pathway analysis (shown below in Figure 4), it seems that the immune system was weakened by I/R injury, but enhanced by the needling at both the PC6 Neiguan and non-acupoint. This kind of nonspecific immune response might contribute to, but not the main mechanism for the protection against I/R injury, though further studies are needed.

These data sets indicate that cardioprotective results delivered from the EA pretreatment may correlate with the gene expressions in the I/R hearts. With pretreatment by EA, many pathological genes in I/R condition were regulated, and the injury of cardiomyocytes was attenuated effectively. NA, acupuncture at non-acupoint might affect some gene expressions as well, but the partial or nonspecific modifications might not be functional enough against I/R injury.

#### 2.2.2. Pathway Analysis

To investigate potential pathways involved in EA or NA treatment to I/R injury, we utilized the Kyoto Encyclopedia of Genes and Genomes (KEGG) pathway analysis from DAVID [33,34]. Pathway analysis was conducted for the DEGs genes among four groups (Figure 4). Figure 4A,B showed the related pathways for the 317 overlapped genes by EA from Figure 3A and the 275 genes from Figure 3C, respectively. Genes included in these pathways are summarized in Supplementary Tables S1 and Tables S2. Other 111 and 127 overlapped genes in NA treatment (Figure 3B,D) were analyzed and shown in Figure 4C,D and Supplementary Tables S3 and S4. Compared with the NA group, the pathological genes regulated by EA include relatively more functional pathways, such as the cardiac muscle contraction pathway (contains Actc1, Myl3, Cyc1, Cox6a1, Myh6, Tnni3, Cacna1c, Cox5b genes), vascular smooth muscle contraction (e.g., Kcnmb4, Actc1, Adora2a, Rhoc, Cacna1c, Pla2g5, Myl9) and hypertrophic cardiomyopathy (e.g., Actc1, Myl3, Myh6, Tnni3, Cacna1c) pathways. On the other hand, the down‑regulated genes are mainly involved in oxidative phosphorylation (e.g., Atp5d, Ndufb7, Ndufb8, Ndufa7, Cyc1, Atp5g2, Atp5g1, Cox5b, Atp5g3, Ndufb2, Ndufs7, Ndufs8, Cos6a1, Atp5l) and focal adhesion (e.g., Cav3, Pgf, Bcar1, Col5a3, Flnc, Col5a1, Myl9, Vegfb, Lama5, Col6a2, Col1a2, Rhoc, Col1a1) pathways.

I/R injury resulted in up- or down-regulation of hundreds of genes. When the rats were subjected to EA pretreatment for two weeks, 44% of the up-regulated and 32% of the down-regulated genes in I/R hearts were reversely modulated. Many of these genes were previously reported to contribute to cardioprotective functions, suggesting electro-acupuncture attenuated I/R injury, at least partially through regulating functional gene expressions. The pathway analysis provided evidence that electro‑acupuncture pretreatment to I/R animals could suppress some functional pathways, such as oxidative phosphorylation, focal adhesion, ECM-receptor interaction, MAPK signaling pathway, Gap junction, vascular smooth muscle contraction, hypertrophic cardiomyopathy, and cardio muscle contraction (Figure 4A). Electro-acupuncture activated some genes involved in apoptosis, NOD-like receptor, cytosolic DNA-sensing pathway, p53, leukocyte transendothelial migration, natural killer cell mediated cytotoxicity, cytokine-cytokine receptor interaction, adipocytokine, and B cell receptor pathways (Figure 4B). Although several common pathways, such as ECM-receptor interaction, MAPK signaling pathway, apoptosis, natural killer cell mediated cytotoxicity, cytokine-cytokine receptor interaction, and leukocyte transendothelial migration pathways were detected in both EA and NA pretreatment groups (Figure 4C,D), most of the genes involved in these pathways were different in EA and NA group; only 27 genes appeared in both EA and NA rats. Moreover, the numbers of genes regulated by EA and NA were largely different, suggesting that the possible mechanisms of cardioprotective effects of EA were dependent on gene regulation patterns.

Cardiac muscle contraction is one of the most important factors in maintaining physiological functions of the heart. Myocardial ischemia injury results in dysfunctional contraction of the left ventricle, which can be exacerbated by reperfusion. Myosin heavy chain (e.g., Myh7b, Myh6) and α-actinin (Actc1) proteins are closely related to cardiac contractile and sarcomeric function [35]. Our results detected that some causative genes, such as Myh7b, Myl3, Tnni3, Actc1, were up-regulated in I/R hearts and were reversed by EA pretreatment. We also found that electro-acupuncture repressed Nppb expression (GSE61840 [36]), suggesting its protection against oxidative stress [37]. Oxidative stress results in changes in cell calcium levels following I/R injury, and contributes to morbidity and mortality [38,39]; calcium influx increases via the L-type calcium channel inducing cardiac diseases [39]. Increased Cacna1c expression (Supplementary Tables S1, GSE61840) in the I/R hearts and its down‑regulation by electro-acupuncture pretreatment suggest that protection by electro-acupuncture against I/R injury is attributed to the expression of this gene. Oxidative stress and calcium channel related pathways contribute to the release of CK and LDH, as well as arrhythmia occurrence after I/R.

Apoptotic pathways were present in both EA and NA cases (Figure 4B,D and Supplementary Tables S2 and Tables S3). Tnfsf10, Xiap, IL1rap, Pik3r1 were involved in both EA and NA, but Chuk was only involved in EA. Surprisingly, we observed that the executioner caspase processing was not enhanced in the I/R group; the apoptotic genes Casp8 and Casp3 were not up-regulated in the I/R hearts, and neither EA nor NA affected its expression (GSE61840). Instead, caspase-independent cell death was activated in I/R in our study (data not shown). Myocardial ischemia reperfusion induces the release of cyochrome c (a pro-apoptotic factor), AIF and Endo G from mitochondria in the absence of caspase activation. Our data showed increased expressions in Cyc1, Endo G, and AIF in myocardial I/R, which were reversed by EA but not NA pretreatment (GSE61840). The anti-apoptotic genes, including Xiap, Bcl2a1d, were up-regulated by electro-acupuncture pretreatment, while they were down‑regulated in I/R hearts. Although the mechanisms by which EA activated mitochondria-mediated anti‑apoptosis and its cardioprotective roles against I/R injury remain unclear, our RNA-seq data analysis demonstrates that EA pretreatment is associated with reduction in apoptotic factors.

Numerous functional pathways and genes are activated or repressed by EA pretreatment in our study. However, further studies are warranted to investigate the role of these important pathways modulated by EA pretreatment applied to myocardial I/R. The placebo treatment by NA included less functional pathways, though it regulated some gene expressions at certain extent. The detailed mechanisms of different gene expression patterns resulting from pretreatment on myocardial I/R injury using acupuncture at acupoint and non-acupoint remain to be investigated.

#### 2.2.3. Confirmation of RNA-seq Data by qRT-PCR

**Figure 5:**
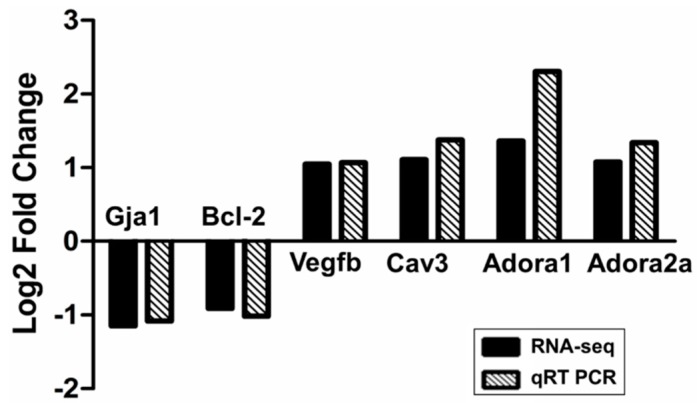
qRT-PCR validation of RNA-seq results. Comparison of fold change (log2) in differential expression values determined by RNA-seq (black) and qRT-PCR (spodoptera) for DEGs.

Transcriptomic data was verified in a randomly selected subset of samples (*n* = 6/each group). Six DEGs were chosen based on RNA-Seq data, including Gja1, Bcl-2, Vegfb, Cav3, Adora1, Adora2a and GAPDH as a reference gene. RT-PCR confirmed that similar tendency as shown in RNA-seq data for five out of six genes. Adora1 showed a much higher log2 fold change using RT-PCR than predicted from RNA-seq data (Figure 5). Therefore, our RNA-seq results must be reliable.

## 3. Experimental Section

### 3.1. Ethics Statement

All animal experiments were conducted at the Laboratory Animal Center of Nanjing University of Chinese Medicine, Nanjing, China. All experimental procedures were approved by the Institute for Animal Care and Use Committee at Nanjing University of Chinese Medicine and conformed to the Guide for the Care and Use of Laboratory Animals [40].

### 3.2. General Reagents

Trihydroxymethylaminomethane (Tris), glycine, sodiumdodecyl sulfate (SDS), acrylamide and bis-acrylamide were purchased from Amresco (Solon, OH, USA). Plasma serum creatine kinase levels (CK), lactate dehydrogenase (LDH), creatinekinase Mb (CK-Mb), and plasma cardiac troponin T (cTnT) detections were performed by the Medical laboratory of Jiangsu Province Hospital (Nanjing, China). Dynabeads protein A was obtained from Invitrogen. Supersignal west picochemiluminescent substrate was purchased from Pierce (Rockford, IL, USA). Truseq RNA sample prep kit-v2, Truseq DNA sample prep kit-PCR box, c-BOT Multiplex re-hybridization plate, and TruseqSbs kit V3 were all purchased from Illumina (San Diego, CA, USA).

### 3.3. Experimental Animals and Groups

**Figure 6:**
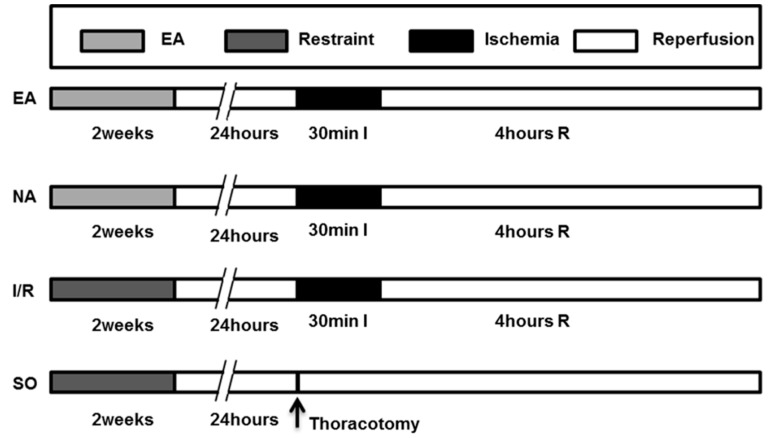
Experimental protocol. See Experimental Section 3.4 for grouping and treatments. EA group (*n* = 15); NA group (*n* = 15); I/R group (*n* = 15); SO group (*n* = 15). EA, electro-acupuncture at PC6 Neiguan acupoint. NA, electro-acupuncture at the base point of the tail. I, ischemia. R, reperfusion.

Male adult Sprague-Dawley (SD) rats (weighing, 280 ± 20 g) were purchased from Vital River Laboratory Animal Technology Co. Ltd (SCXK 2008-0004, Beijing, China). The rats were kept under constant temperature (25 ± 2 °C), constant relatively humidity of 50% ± 5% and normal photoperiod (12 h light/12 h dark). The experimental protocols are depicted in Figure 6. The 60 rats were randomly assigned to four groups, and 15 rats in each one: (1) SO: the LAD was exposed but not occluded, no treatment applied; (2) EA: PC6Neiguanacupoint was electrically stimulated for 2 weeks before operation; (3) NA: Non-acupoint (base of tail) was electrically stimulated for 2 weeks before operation; (4) I/R: underwent operation of ischemia-reperfusion but without electro-acupuncture treatment.

### 3.4. In Vivo Myocardial Ischemia-Reperfusion

The animals were anesthetized with 5% isoflurane followed by continuous inhalation of 1%–2% isoflurane in a mix of 70% N2O and 30% O2, then intubated and mechanically ventilated with room air using a rodent respirator (RWD Life Science Co. Ltd. (Shenzhen, China), 45–60 breaths per minute, and tidal volume was set to 1.0 mL/100 g body weight). Under anesthesia a left thoracotomy was carried out to expose the heart between the fourth and fifth intercostal space. Following pericardiotomy, the left anterior descending coronary artery (LAD) was occluded with a 6.0 silk suture for 30 min ischemia followed by 4 h of reperfusion [41]. The chest was closed 30 min after the LAD was reperfused, and the animals were allowed to recover. Given the acute experiment, after the operation buprenorphine HCl (0.05 mg/kg) was administered immediately by intramuscular injection to minimize pain and distress [42]. A Lead II ECG was successive monitored and recorded under anesthesia from 30 min before operation to 30 min after post-reperfusion, then monitored once again from 210 min to 240 min after reperfusion under re-anesthesia. The blood samples and heart tissue were collected after ECG recording was completed under anesthesia with isoflurane (5%). Successful occlusion was confirmed by the development of a cyanotic anterior ventricular wall, a significant ST segment elevation, and a peaked T-wave on the ECG (A standard limb lead II electrocardiogram). During the process, the body temperature was maintained by rectal thermometer and maintained constant between 37.1 and 37.5 °C by a heating pad. For survival study, mortality rate was recorded every 0.5 h for up to 4 h after the operation.

### 3.5. Restraint and Electro-Acupuncture Intervention

Prior to myocardial ischemia-reperfusion experiment, all rats were restrained in tubes for 20 min daily for 12 days. In addition, the animals in both EA (*n* = 15) and NA (*n* = 15) groups were pretreated with electro-acupuncture for 20 min daily for total of 12 days based on restraint, with a day of rest after six days of treatment. The PC6 Neiguan acupoint is located in the forelimbs according to the textbook of experimental acupuncture in animals [8]. Two acupuncture needles (Gauge-28, 0.5 cm) were separately inserted into the PC6 Neiguan acupoint on each limb, and an electrical current was provided to the needles through an electrical stimulator for 20 min, with a stimulus isolation unit (Han’s acupoint nerve stimulator, HANS-200, Nanjing, China) at a frequency of 2/15 Hz and an intensity level of 1 mA [43]. The intensity was adjusted to induce a slight repetitive flexion of the paw which could be observed during electrical stimulation [44,45].The acupuncture needle, 15 mm long and 0.3 mm in diameter, was inserted 2–3 mm into the subcutis. The same pretreatments were applied to the NA animals at the base of the tail. The acupuncture procedure was carried out with extremely gentle operation to avoid any unnecessary stimulus and stress to the rats.

### 3.6. Arrhythmia Score

The arrhythmias were assessed during a period of 30 min of ischemia followed by 30 min of reperfusion, and arrhythmia inductions by I/R at 240 min were not found. There were seven rats that died in first 30 min of reperfusion (two in I/R, three in EA, and two in NA group, respectively. We scored arrhythmias in survived animals (15 in SO, 13 in I/R, 12 in EA, and 13 in NA) according to the system by Curtis and Walker [6]: 0 = no arrhythmia; 1 ≤ 10 s premature ventricular contraction (VPC ) and/or ventricular tachycardia (VT); 2 = 11–30 s VPC and/ or VT; 3 = 31–90 s VPC and/ or VT; 4 = 91–180 s VPC and/or VT, or reversible ventricular fibrillation (VF) of ≤10 s; 5 ≥180 s VPC and/or VT, >10 s reversible VF; 6 = irreversible VF.

### 3.7. Infarct Size

At the end of experiment, 240 min reperfusion, under anesthesia the heart was promptly removed from SO (*n* = 5), I/R (*n* = 4), EA (*n* = 5) and NA (*n* = 5) group and stored in −20 °C. It was then cut transversely into five to six slices of equal thickness (about 1–2 mm) from the apex to the base. The slices were incubated at 37 °C in 1% solution of buffered (pH 7.4) triphenyltetrazolium chloride (TTC, Sigma-Aldrich, St. Louis, MO, USA) for 10 min to expose the area of infarct [46]. The infarct area was expressed as a percentage of the left ventricular mass, which was quantified with Image J software [47].

### 3.8. Measurement of Serum Levels of CK, LDH, CK-Mb, Plasma Level of cTnT

Following a four-hour reperfusion, under anesthesia blood samples were obtained and centrifuged from SO (*n* = 15), I/R (*n* = 6), EA (*n* = 11) and NA (*n* = 8) group. Serum LDH, CK and CK-Mb were analyzed by immune inhibition, and plasma cTnT was detected with electrochemiluminescence immune-assay method at the Medical Laboratory of Jiangsu Province Hospital (Nanjing, China).

### 3.9. RNA-seq

Total RNAs were extracted by Trizol reagent (Invitrogen, Mannheim, Germany) from the harvested left ventricles (*n* = 2, each group). Concentration and purity of RNA were determined by automated optical density evaluation (OD 260/OD 280 ≥ 1.8 and OD 260/OD 230 ≥ 1.8) using Nanodrop ND‑1000 (Nanodrop Technologies, Wilmington, DE, USA). The degree of RNA degradation was analyzed by the Agilent electrophoresis bioanalyzer 2100 (Agilent Technologies Inc., Santa Clara, CA, USA) with the RNA integrity number values consistently above 8 (Supplementary Figure S2). RNA samples were prepared according to the TruSeq RNA Sample Preparation v2 protocol, and the DNA libraries were applied to the cluster generation and sequencing using c-BOT Multiplex re-hybridization plate and TruseqSbs kit V3.The final product should be a band at approximately 260 bp (for single‑read libraries). Sequencing was performed using Illumina HiSeq 2000 (Illumina).

### 3.10. Computational Analysis for RNA-seq Data

After sequencing with HiSeq 2000 (Illumina), raw fastq files were extracted from Illumina BCL using the Illumina CASAVA program. The single-end reads obtained from each sample were aligned to the rat reference genome (UCSC rn4 assembly) using the TopHat [48,49]. The Cufflinks program was used to assemble individual transcripts from the RNA-seq reads that have been aligned to the genome and to qualify the expression level of each transcript. Differential transcripts expression analysis was performed using the Cuffdiff program [50], we used two cutoff values; unadjusted *p*‑value and fold-change cutoffs. The gene’s functional annotation and pathway were analyzed using the DAVID Bioinformatics Resources [51]. The raw RNA-seq data were uploaded to GEO (GSE61840). Genes with lower than 1 FPKM (average fragments per kilobase of transcript per million fragments mapped) were filtered out. Up-regulation and down-regulation were defined as a relative transcription level above Log2 fold change (FC) ≥ |±1|. The number of mapped reads was more than 90% of the total reads (Supplementary Tables S5).

### 3.11. qRT-PCR

Four micrograms of RNA were converted to cDNA using reverse transcriptase and random primers (#1621, Thermo, Waltham, MA, USA). The primer sequences were shown in Supplementary Tables S6. For PCR analysis, the samples were amplified in duplication using SYBR Green (Thermo, #PC4602), with 200 nM of gene-specific primers and run on the CFX amplifier (MX3000P, Stratagene, La Jolla, CA, USA) using the manufacturer’s protocol. Data were analyzed by threshold cycle (Ct) relative-quantification method.

### 3.12. Statistics Analysis

For statistics purposes, all data are expressed as means ± standard error of the means and were analyzed using SPSS16.0 (SSPS Inc., Chicago, IL, USA). A one-way analysis of variance (ANOVA) is used for group comparison, and the Student-Newman-Keuls test is used for multiple comparisons. A *p*-value derived from a two-tailed test less than 0.05 was considered significant.

## 4. Conclusions

In summary, our data indicate that pretreatment using electro-acupuncture at the PC6 Neiguan acupoint exerts a cardioprotective effect in I/R rats through modulating functional gene expressions. This study provided, for the first time, informative genome-wide profiles of gene expressions in I/R injury and electro-acupuncture pretreatment that can be used in future functional studies. Compared to non-acupoint pretreatment, needling at the PC6 Neiguan acupoint specifically regulated cardiac muscle contraction, vascular smooth muscle contraction, hypertrophic cardiomyopathy, oxidative phosphorylation, inflammation and immune response, and apoptosis pathways, thus effectively protected against I/R injury in a pretreatment approach. This study also provided supportive evidences that acupuncture at the PC6 Neiguan acupoint might be a useful therapy prior to the application of reperfusion to patients with myocardial ischemia.

## Supplementary Materials

Supplementary materials can be accessed at: http://www.mdpi.com/1420-3049/19/10/16158/s1.

## Supplementary Files

Supplementary File 1
