# Supplementary material for: Electro-Acupuncture at Neiguan Pretreatment Alters Genome-Wide Gene Expressions and Protects Rat Myocardium against Ischemia-Reperfusion
Source: Molecules. 2014 Oct 9;19(10):16158–78. doi: 10.3390/molecules191016158 (PMC6271995; doi:10.3390/molecules191016158)
Supplement: Supplementary File 1 [file molecules-19-16158-s001.pdf]

# Supplementary Materials

**Figure S1.** Multidimensional scaling (MDS) plot, MDS plot of samples based on genes show the relative similarities of samples to differentially expressed among four groups.

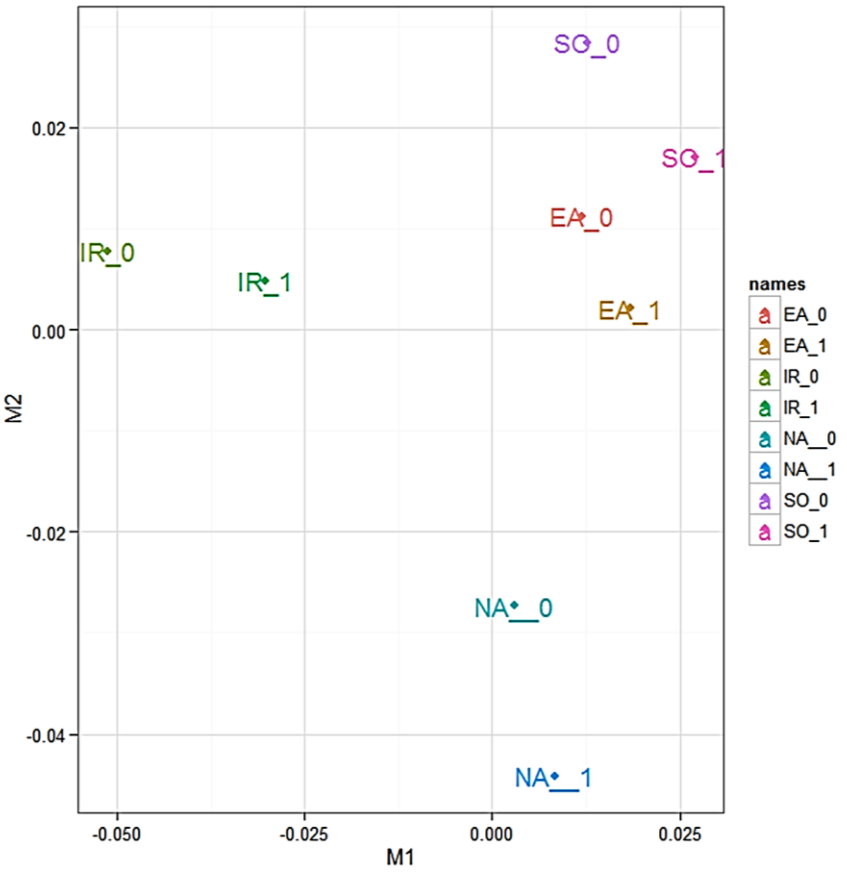


**Figure S2.** RNA integrity number (RIN) value.

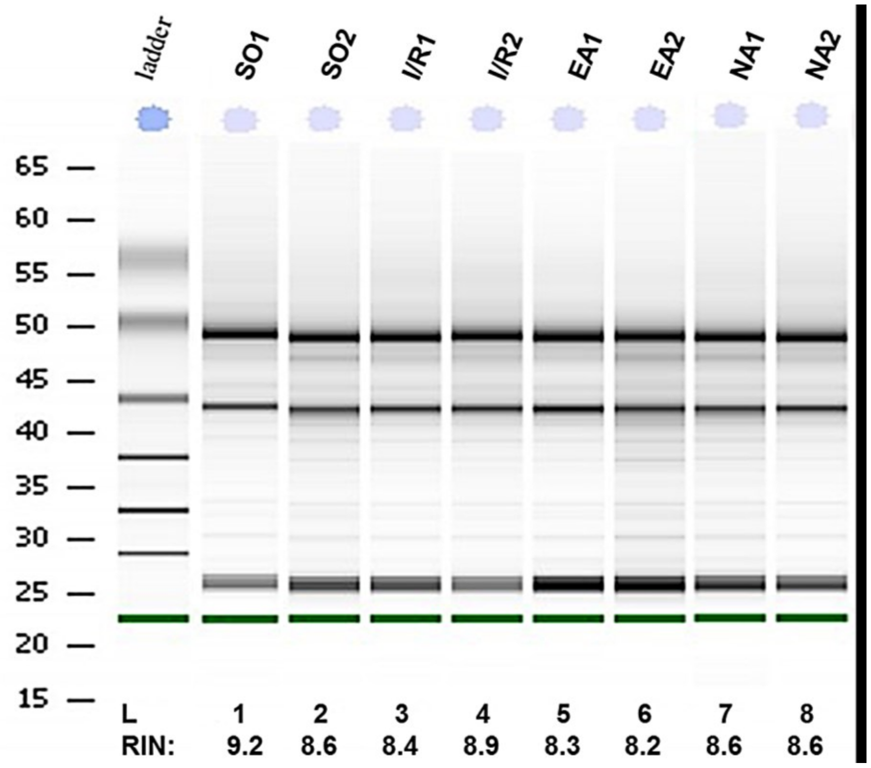

**Table S1.** Genes up-regulated in I/R group and down-regulated in EA group.

| Pathway | Genes |
|---|---|
| Parkinson’s disease | SEPT5, ATP5D, NDUFB7, NDUFB8, NDUFA7, CYC1, ATP5G2, ATP5G1, COX5B, ATP5G3, VDAC3, NDUFB2, NDUFS7, GP1BB, NDUFS8, COX6A1 |
| Oxidative phosphorylation | ATP5D, NDUFB7, NDUFB8, NDUFA7, CYC1, ATP5G2, ATP5G1, COX5B, ATP5G3, NDUFB2, NDUFS7, NDUFS8, COX6A1, ATP5L |
| Huntington’s disease | ATP5D, NDUFB7, NDUFB8, NDUFA7, CYC1, ATP5G2, ATP5G1, COX5B, ATP5G3, VDAC3, NDUFB2, NDUFS7, NDUFS8, COX6A1 |
| Alzheimer’s disease | ATP5D, NDUFB7, NDUFB8, NDUFA7, CYC1, ATP5G2, ATP5G1, COX5B, ATP5G3, NDUFB2, NDUFS7, NDUFS8, COX6A1, CACNA1C |
| Focal adhesion | CAV3, PGF, BCAR1, COL5A3, FLNC, COL5A1, MYL9, VEGFB, LAMA5, COL6A2, COL1A2, RHOC, COL1A1 |
| Cardiac muscle contraction | ACTC1, MYL3, CYC1, COX6A1, MYH6, TNNI3, CACNA1C, COX5B |
| ECM-receptor interaction | SEPT5, GP1BB, LAMA5, COL1A2, COL6A2, COL1A1, COL5A3, COL5A1, SDC3 |
| MAPK signaling pathway | MAP2K3, HSPA1B, FLNC, CDC25B, DUSP5, DUSP14, GADD45G, JUND, HSPB1, CACNA1C, NFATC2, PLA2G5 |
| Gap junction | TUBB5, TUBB6, TUBA4A, TUBA1B, TUBA1C, TUBB3 |
| Vascular smooth muscle contraction | KCNMB4, ACTC1, ADORA2A, RHOC, CACNA1C, PLA2G5, MYL9 |
| Hypertrophic cardiomyopathy | ACTC1, MYL3, MYH6, TNNI3, CACNA1C |

**Table S2.** Genes down-regulated in I/R group and up-regulated in EA group.

| Pathway | Genes |
|---|---|
| NOD-like receptor signaling pathway | CCL12, XIAP, IL18, CASP1, CARD6, CHUK |
| Cytosolic DNA-sensing pathway | TBK1, IL18, IL33, CASP1, CHUK |
| p53 signaling pathway | CCNE2, IGF1, RRM2B, PTEN, SESN3 |
| Leukocyte transendothelial migration | VCAM1, CYBB, GNAI1, RAP1B, JAM2, PIK3R1 |
| Small cell lung cancer | CCNE2, XIAP, PTEN, CHUK, PIK3R1 |
| Apoptosis | TNFSF10, XIAP, IL1RAP, CHUK, PIK3R1 |
| Prostate cancer | CCNE2, IGF1, PTEN, CHUK, PIK3R1 |
| Natural killer cell mediated cytotoxicity | CD48, TNFSF10, CD244, FCGR2B, PIK3R1 |
| Cytokine-cytokine receptor interaction | TNFSF10, CXCL13, IL18, IL1RAP, LIFR, KITLG, IL13RA1 |
| Long-term depression | GNAQ, GNAI1, IGF1, GUCY1B3 |
| Adipocytokine signaling pathway | CD36, ACSL4, ACSL3, CHUK |
| B cell receptor signaling pathway | FCGR2B, RASGRP3, CHUK, PIK3R1 |

**Table S3.** Genes down-regulated in I/R group and up-regulated in NA group.

| Pathway | Genes |
|---|---|
| Apoptosis | TNFSF10, XIAP, IL1RAP, PIK3R1 |
| Natural killer cell mediated cytotoxicity | TNFSF10, CD244, FCGR2B, PIK3R1 |
| Cytokine-cytokine receptor interaction | TNFSF10, CXCL13, IL1RAP, LIFR, IL13RA1 |
| Leukocyte transendothelial migration | VCAM1, CYBB, RAP1B, PIK3R1 |
| Renal cell carcinoma | HIF1A, RAP1B, PIK3R1 |

**Table S4.** Genes up-regulated in I/R group and down-regulated in NA group.

| Pathway | Genes |
|---|---|
| ECM-receptor interaction | SEPT5, GP1BB, COL1A1, COL5A3, COL5A1 |
| Parkinson's disease | SEPT5, GP1BB, COX5B, VDAC3, NDUFB2 |
| MAPK signaling pathway | FOS, JUND, NR4A1, FLNC, NFATC2 |
| Keratan sulfate biosynthesis | B3GNT7, CHST1 |

**Table S5.** Number of total reads and mapped reads.

| Group | Fastq_Name | Total Reads | Mapped Reads | % |
|---|---|---|---|---|
| SO_0 | 11_ACTTGA_L002_R1_001.fastq | 39862003 | 37318902 | 93.62 |
| SO_1 | 12_GGCTAC_L003_R1_001.fastq | 40442143 | 37702209 | 93.23 |
| IR_0 | 13_TAGCTT_L002_R1_001.fastq | 30662110 | 28700545 | 93.60 |
| IR_1 | 14_GTGGCC_L003_R1_001.fastq | 43356297 | 40111978 | 92.52 |
| EA_0 | 15_GGCTAC_L002_R1_001.fastq | 41608188 | 38659922 | 92.91 |
| EA_1 | 16_CGTACG_L002_R1_001.fastq | 41844123 | 38578194 | 92.20 |
| NA_0 | 17_GTGGCC_L002_R1_001.fastq | 35675554 | 33309894 | 93.37 |
| NA_1 | 18_CGTACG_L003_R1_001.fastq | 20577495 | 19105240 | 92.85 |

**Table S6.** Primer sequences for Real-time PCR.

| Gene | Forward Primer (5'-3') | Reverse Primer (5'-3') | Tm (°C) | Product (bp) |
|---|---|---|---|---|
| Gja1 | CACTAGCCATTGTGGACCAG | CTAGATCTCCAGGTCATCAGG | 58 | 158 |
| Vegfb | CGCGGATCCGCCCCTGTCTCCCAGCCTG | CCGGAATTCTAAGCCCCGCCCTTGGCAAC | 60 | 200 |
| Bcl-2 | AACATCGCCCTGTGGATGACTG | GCTGATTCGACGTTTTGCCTGA | 56 | 112 |
| Cav3 | GCTGATTCGACGTTTTGCCTGA | GCTGATTCGACGTTTTGCCTGA | 62 | 137 |
| Adora1 | TACATTGGCATCGAGGTCCTC ATT | GAGCTCTGGGTGAGGATGAGGAC | 54 | 196 |
| Adora2a | TCAGAATTCTAATGGTITACTAA | TTCAAGCTTGGIACCAIAIGCAA | 57 | 129 |
| GAPDH | TGCCCAGAATATCATCCC | AGGTCAGATCCACAACAG | 59 | 134 |
